# Improving Adolescent Care in a Cross-Sector System

**DOI:** 10.1192/bjo.2022.334

**Published:** 2022-06-20

**Authors:** Aarthi Ravishankar, Tom Holliday, Lauren Fraser, James Biggin-Lamming

**Affiliations:** London North West University Healthcare NHS Trust, London, United Kingdom

## Abstract

**Aims:**

Adolescence represents a critical life stage in which there is rapid physical, cognitive and psychosocial development. It is the time where the patterns and foundations for future health are laid and thus presents a unique opportunity to promote health and subsequently improve life-long well-being and reduce health inequalities. Mental health problems represent the greatest contributors to disease burden for this population and this contribution is forecast to rise. The World Health Organisation state that adolescents and young adults (AYAs) need health services that are supportive, equitable and effective. The project aims to scope out Adolescent care at London North West University Healthcare NHS Trust (LNWH) with a view to improve quality of care for this group of patients.

**Methods:**

Quantitative data obtained assessed patterns of presentation to the Emergency department (ED). Qualitative data were obtained through stakeholders Interviews with professionals, adolescent patients and their caregivers. As of January 2022, 113 stakeholders were interviewed. The data obtained informed the creation of the ‘LNWH AYA Manifesto.’ This was converted into a questionnaire for all professionals involved in the care of AYA patients to assess organisational culture around AYA Care.

**Results:**

It was found that AYA care at LNWH lies across a complex cross-sector system. The commonest code for presentation to the ED for those ages 13 to 25 was ‘depressive disorder’. Key themes from stakeholder interviews included: 1) AYAs are not always provided with age-appropriate care 2) Acute Trusts may serve as a catalyst for change for AYA patients and Youth workers may be better placed to connect with them 3) There is a need for an integrated approach to physical and mental health with better relationships needed between the Acute teams and CAMHS. The ‘LNWH AYA Manifesto’ questionnaire found disparate opinions regarding the approach to integrated physical and mental health; of the 34 responses obtained 23.5% reported not feeling confident with recognising and managing mental health and social issues in AYAs and 41.1% believed that physical and mental health problems should be addressed separately by the relevant specialties.

**Conclusion:**

AYA care lies across a complex cross-sector system and thus requires a multifactorial approach to create a culture change towards prioritising this population. One such intervention proposed is the introduction of a Youth Worker outreach model similar to the King's Adolescent Outreach Service as a way to create a shift towards an integrated approach to physical and mental health care.

